# Current Therapy of the Patients with MDS: Walking towards Personalized Therapy

**DOI:** 10.3390/jcm10102107

**Published:** 2021-05-13

**Authors:** Maria Luisa Palacios-Berraquero, Ana Alfonso-Piérola

**Affiliations:** Hematology and Hemotherapy Department, Clínica Universidad de Navarra, 31008 Pamplona, Spain; mpalaciosb@unav.es

**Keywords:** myelodysplastic syndrome, treatment, luspatercept, azacitidine, pevonedistat, magrolimab, eprenetapopt, venetoclax

## Abstract

Myelodysplastic syndromes (MDS) are characterized by ineffective hematopoiesis, dysplasia and peripheral cytopenias. Nowadays, MDS therapy is selected based on risk. The goals of therapy are different in low-risk and high-risk patients. In low-risk MDS, the goal is to decrease transfusion needs and to increase the quality of life. Currently, available drugs for newly diagnosed low-risk MDS include growth factor support, lenalidomide and immunosuppressive therapy. Additionally, luspatercept has recently been added to treat patients with MDS with ring sideroblasts, who are not candidates or have lost the response to erythropoiesis-stimulating agents. Treatment of high-risk patients is aimed to improve survival. To date, the only currently approved treatments are hypomethylating agents and allogeneic stem cell transplantation. However, the future for MDS patients is promising. In recent years, we are witnessing the emergence of multiple treatment combinations based on hypomethylating agents (pevonedistat, magrolimab, eprenetapopt, venetoclax) that have proven to be effective in MDS, even those with high-risk factors. Furthermore, the approval in the US of an oral hypomethylating agent opens the door to exclusively oral combinations for these patients and their consequent impact on the quality of life of these patients. Relapsed and refractory patients remain an unmet clinical need. We need more drugs and clinical trials for this profile of patients who have a dismal prognosis.

## 1. Introduction

Myelodysplastic syndromes (MDS) comprise a group of heterogeneous myeloid malignancies that bear in common the presence of cytopenias due to ineffective hematopoiesis and an increased risk of acute myeloid leukemia transformation [1]. They mainly affect an older population, displaying complex pathogenesis and diverse natural histories. The prognosis of MDS patients is extremely heterogeneous. Currently, the standard tools for risk assessment are the International Prognostic Scoring System (IPSS) or the Revised International Prognostic Scoring System (IPSS-R) [2,3] (Table 1), both of them based on the number of cytopenias, the percentage of blasts and the karyotype. New prognostic scales with more weight on molecular aspects that consider their impact on prognosis are needed to refine our prognostic ability and better guide treatment strategies at an individual level [4].

At present, risk-adapted therapy is used in patients with MDS. Patients with MDS are divided into two groups regarding their IPSS or IPSS-R: low- and high-risk disease. Low-risk MDS includes patients with low- and intermediate-1-risk by IPSS and/or very low, low- and some subsets of intermediate-risk by IPSS-R. High-risk MDS includes patients with intermediate-2- and high-risk disease by IPSS and some subsets of intermediate-, high- and very high-risk disease by IPSS-R [2,3].

However, despite great advances in the prognostic estimation of patients with MDS, approved treatment options are currently scarce. In recent years there has been an outburst of new investigational approaches with new therapeutical targets. In this review, we describe the current therapies available for MDS patients and the potential new drugs that are under investigation.

## 2. Lower-Risk MDS (LR-MDS)

The aim of therapy in this subset of patients is to improve the symptoms due to cytopenia, decrease transfusion needs, improve quality of life, attempt to reduce the risk of progression and, ultimately, improve survival.

### 2.1. Newly Diagnosed Patients with LR-MDS

#### 2.1.1. Erythroid Stimulating Agents

Erythropoiesis stimulating agents (ESA) are currently the first choice for treating anemia in low-risk MDSs and have thus been extensively used in Europe for the last decades. Several ESA are available with reported response rates ranging from 30% to 60%, according to studies [5].

The validated Nordic scoring system showed that LR-MDS patients with serum erythropoietin (EPO) < 100 U/L and a transfusion requirement of <2 packed red blood cells per month have a greater than 70% probability of responding to ESA, unlike those with EPO > 500 U/L that have an estimated response rate of less than 10% [6].

Recently, two major phases III clinical trials comparing darbepoetin and epoetin alfa versus placebo have established the effectiveness of ESA on erythroid response and reduction in transfusion incidence, specifically in patients with MDS. The first randomized, placebo-controlled, phase III study of darbepoetin alfa included 147 lower/intermediate-1 risk MDS patients. It showed higher erythroid responses (14.7% vs. 0%, *p* = 0.016) with a reduction in transfusion dependence in patients treated with darbepoetin compared to placebo (59% vs. 36%, *p* = 0.008), with no significant rise in the incidence of thromboembolic events [7]. Another double-blind, placebo-controlled trial included 130 low/intermediate-1 risk patients randomized to receive epoetin-alpha or placebo. There were significantly higher erythroid responses (31.8% for epoetin-alpha vs. 4.45 placebo, *p* = 0.001), a reduction in total transfusions and an increased time-to-first transfusion (median 7.0 vs. 5.3 weeks, respectively *p* = 0.046) compared with placebo [8]. The median response duration was of 40 weeks. Interestingly, there were no significant differences in quality-of-life (QoL) assessments between both groups at any time point, but only between responders and nonresponders independent of therapy. As a result of these studies, erythropoietin alpha has been approved in Europe specifically to treat MDS.

An important fact about ESA is that high doses of ESA have greater erythroid responses (for both epoetin-alpha and darbepoetin) than those receiving standard doses [5].

There is still question about the addition of granulocyte colony-stimulating factors (G-CSF) to increase response to ESA, partly because of the lack of available data. Several studies point towards increased response rates and an impact on survival with the early introduction of ESA combined with G-CSF [9,10,11]; however, data are still controversial.

The French group, in a retrospective study with 284 patients, evaluated the impact of ESA on survival compared to a historical cohort [11]. There was significantly better survival (HR for death: 0.43, 95% CI 0.25–0.72) in patients treated with ESA. However, to date, no randomized prospective study has proven that treatment with ESA impacts survival in patients with MDS.

Recommendations: We recommend using ESA in those patients with low-risk MDS and hemoglobin below 10 g/dL, who do not have both predictors of poor response to these agents (≥2 packed red blood cells per month and endogenous erythropoietin levels ≥500 IU/L). It is advisable to start treatment with high doses to optimize treatment [12].

#### 2.1.2. Thrombopoietic Agents

The success in treating anemia in MDS with ESA has piqued interest in using thrombopoiesis stimulating agents, particularly thrombopoietin agonists (TPO), leading to several studies investigating their effectiveness.

Romiplostim is a recombinant peptibody that targets the thrombopoietin (TPO) receptor to increase platelet production and is approved for use in adults with chronic immune thrombocytopenia. The use of romiplostim in patients with lower-risk MDS has been explored in an extension study that investigated its use in monotherapy in 60 patients with grade 3 thrombocytopenia (platelet count of <50 × 10^9^/L) [13]. 52% achieved platelet response, with 82% presenting a sustained response per International Working Group (IWG) 2006 criteria, with a median response duration of 52 weeks. Bleeding events were significantly lower among responders, while only four in this subset of patients had thromboembolic events.

The efficacy of eltrombopag is currently under investigation. The largest study is a single-blind, randomized, controlled phase II superiority trial [14] of adult patients with low-risk or IPSS intermediate-1-risk myelodysplastic syndromes and severe thrombocytopenia (platelet count <30 × 10^9^/L). Platelet responses occurred in 47% of patients treated with eltrombopag versus 3% in the placebo group (*p* = 0.002). There were a higher number of bleeding events in the placebo than in the eltrombopag arm (42% vs. 14% *p* = 0.0025), without significant differences in AML evolution or disease progression (12% vs. 16%, *p* = 0.81).

Overall, using TPO agonists seems to be clinically effective in increasing platelet counts and reducing bleeding events. However, assessing the long-term safety and efficacy of these agents and their effect on survival has yet to be evaluated. Thus, to date, their use remains exclusive to the setting of clinical trials [15].

Recommendations: The use of thrombopoietic agents (romiplostim, eltrombopag) as supportive treatment in low-risk MDS is not approved, but both have shown efficacy and can be used safely, although always better in the setting of clinical trials and in patients without an excess of blasts [12].

#### 2.1.3. Lenalidomide

Although patients with del(5q) ESA can achieve notable response rates, the duration of these responses is shorter than in other MDS subtypes [16]. Lenalidomide has demonstrated its utility in reducing transfusion requirements and reversing cytologic and cytogenetic abnormalities in patients with a 5q- MDS [17]. 5q deletion leads to haploinsufficiency of *RPS14* and *CSNK1A1*. The reduced expression of *RPS14* affects the biology of the ribosomes, which leads to an accumulation of other components, such as *RPL11*, which sequester MDM2, leading to a decrease in the proteasomal destruction of p53 and increased p53-mediated apoptosis of erythroid progenitors [18,19]. On the other hand, *CSNK1A1* encodes casein kinase 1α (CK1α), negatively regulating the Wnt/β-catenin and p53 pathways. Thus, the haploinsufficiency of *CSNK1A1* leads to increased proliferation and apoptosis of the bone marrow progenitors. Lenalidomide binds CRBN, which recruits and degrades CK1α and leads to p53-dependent apoptosis of cells harboring del(5q) [20].

Different studies have confirmed the efficacy of lenalidomide in patients with anemia and chromosome 5 alterations. A randomized phase III study compared lenalidomide to placebo in 205 transfusion-dependent patients with low/intermediate-risk MDS with del(5q) [21]. More patients in the lenalidomide groups (10 and 5 mg doses) achieved red blood cell (RBC) transfusion independence (TI) compared to placebo (56.1% and 42.6% vs. 5.9%; both *p* = < 0.001). With a median follow-up of 1.55 years, the median duration of RBC-TI was not reached, with 60% to 67% of responses ongoing. Higher doses of lenalidomide (10 mg) were associated with higher response rates (50%) when compared to the lower 5 mg dosage (25%; *p* = 0.066). The 3 year overall survival (OS) and AML risks were 56.5% and 25.1%, respectively, for both groups combined. Of note, patients that achieved RBC-TBI for over 8 weeks had a reduction in the relative risk of death (47%) and AML transformation (42%). Factors significantly predictive of transfusion independence were lenalidomide treatment (*p* < 0.0001 for 10 mg vs. placebo; *p* = 0.0004 for 5 mg vs. placebo), longer time from MDS diagnosis (>2 years; *p* = 0.05) and higher baseline platelet count (≥150 × 10^9^/L; *p* = 0.003). Hematologic toxicity (neutropenia and thrombopenia) was the most common adverse event, with no significant differences between doses. Overall, and supporting the evidence already available from the MDS-003 study [16], lenalidomide treatment can achieve remarkable erythroid and cytogenetic responses, which are durable and are related to improved QoL and reduced risk of death. 10 mg dose can achieve deeper responses without a significant increase in toxicity.

Several studies have tried to assess whether *TP53* mutations can predict poorer outcomes to lenalidomide treatment in terms of response and disease progression. Various have shown a lower probability of a complete cytogenetic response to lenalidomide in *TP53* mutated patients [22,23], with other factors for poor response being a low baseline platelet count and karyotypic complexity. Also, variant allele frequency (VAF) appears to be predictive of outcome in patients with TP53 mutations with remarkably low OS in patients with VAF > 40% [24]. Thus, the molecular study of *TP53* in del(5q) MDS patients is useful before therapy with lenalidomide, and patients harboring any *TP53* alterations should be closely followed to promptly identify treatment failure, refractoriness and progression.

On the other hand, using lenalidomide in patients without chromosome 5 alterations remains the subject of investigation. The most robust evidence comes from 239 transfusion-dependent lower-risk non-del(5q) MDSs ineligible for or refractory to ESA patients in phase III, randomized, placebo-controlled study. Patients receiving lenalidomide achieved higher rates of RBC-TI compared to placebo (26.9% vs. 2.5%, *p* = 0.001), with a median duration of 30.9 weeks. Higher response rates were observed in patients with lower baseline endogenous erythropoietin ≤ 500 mU/mL (34.0% vs. 15.5%). TI leads to a significant improvement in health-related quality of life (HRQoL). In a subset of non-del(5q) MDSs transfusion-dependent patients with resistance o ESA, another phase III randomized trial compared lenalidomide alone and lenalidomide in combination with erythropoietin (EPO) as second-line treatment [25]. There was a significant improvement in erythroid responses when using combined treatment, but not in response duration.

Recommendation: Lenalidomide should be considered the treatment of choice in patients with MDS with 5q deletion and transfusion dependence with a low probability of response to ESA or in whom this treatment has failed. The use of lenalidomide can be considered in selected cases without a 5q deletion. We recommend studying *TP53* mutational status in patients with del5q MDS. No evidence supports withholding therapy with lenalidomide in *TP53* mutated patients. However, these patients should be monitored closely for signs of lack of response or progression [12].

#### 2.1.4. Iron Chelation

Patients with a high RBC transfusion burden may accumulate excess iron, which may cause tissue injury via generating reactive free radicals. However, the impact of iron chelation therapy (ICT) in patients with MDS and high RBC transfusion rate is still poorly understood and has not been extensively studied. A systematic review and meta-analysis of nine non-randomized trials showed a significant reduction in risk of mortality in low or intermediate-risk MDS patients with iron overload treated with ICT. However, no causal relationship has been established [26]. In this sense, the TELESTO trial was the first multicenter, randomized, double-blind, placebo-controlled trial with deferasirox for iron chelation. Initially designed as a phase III trial, it was finally carried out as a phase II study, including 210 patients with event-free survival as the primary endpoint [27]. This included echocardiographic evidence of cardiac function impairment, hospitalization for congestive heart failure, worsening of liver function, cirrhosis, and transformation into acute myeloid leukemia. Events were reduced by 36.4% in the intervention group (HR = 0.636, *p* = 0.01). The outcome of secondary endpoints, such as OS and changes in serum ferritin levels, were consistent with the clinical benefit of deferasirox, with acceptable toxicity. However, more studies are needed to determine the appropriate threshold for treatment initiation.

Recommendation: The current recommendation is to perform iron chelation in patients with MDS who receive periodic transfusion treatment and have a reasonable life expectancy (at least one year) and in candidates for hematopoietic transplantation [12].

#### 2.1.5. Immunosuppressive Therapy

The role of immune therapy and, specifically, immunosuppressive drugs is still controversial. There is mounting evidence that abnormal activation of innate immune signals and associated inflammation play an important part in the biology of MDS, being a particularly relevant pathogenic route in certain subtypes of the disease [28]. In this sense, several studies point towards its applicability in a subset of patients with hypoplastic MDS, HLA-DR15, Trisomy 8, young age (<60) and low transfusion burden [28,29].

A phase 3 clinical trial comparing horse anti-thymocyte globulin (ATG) plus oral cyclosporine (CsA) versus best supportive care (BSC) in MDS reported an overall response rate (ORR) in the ATG arm of 29% with a median duration of response of 16.4 months [30]. Hypoplastic MDS had a higher ORR at 50% in this study, although no significant OS or AML-free survival difference was found between IST vs. BSC arms. A recent meta-analysis showed an overall response rate of 42.5% and a Ran BC-TI rate of 33.4% [31], with a lack of homogeneous reporting of OS and Es. As recently highlighted by the MDACC group [15], younger patients with severe hypoplastic MDS, when found appropriate candidates, should not delay allo-stem cell transplants (allo-SCT). Those not eligible may attempt combined therapy based on antitimoglobulin (ATG), although its frequent associated toxicity excludes most older patients from its use.

Other strategies, such as alemtuzumab monotherapy, have been used in a phase I/II clinical trial, in which 22 patients received 10 mg/day intravenous therapy for 10 days. ORR was 77%, with a median time to response of 3 months [32].

Recommendation: Indications for immunosuppressive therapy in low-risk MDS are currently very limited and should be reserved for patients who have failed other previous lines of treatment and have a high probability of response. Immunosuppressive treatment in LR-MDS should be based on using ATG associated or not with CsA [12].

### 2.2. Relapsed/Refractory LR-MDS

#### 2.2.1. Luspatercept

Luspatercept is a fusion protein that blocks transforming growth factor-beta (TGF β) superfamily via SMAD2 and SMAD3 signaling, consequently allowing erythroid maturation [33,34].

In the phase 2 PACE-MDSs study, 58 LR-MDSs patients were enrolled. Among the patients treated at higher doses (luspatercept 0.75–1.75 mg/kg subcutaneously every 21 days), the study showed a 63% of erythroid response rate, with 38% of the patients achieving transfusion independence [35]. Responses were higher in those patients with SF3B1 mutations (77% vs. 40%, respectively), leading to a phase 3 trial focused on LR-MDS patients with ≥15% ring sideroblast (RS) (or ≥5% RS plus *SF3B1* mutation), who were transfusion-dependent with disease refractory to or unlikely to respond to ESA (MEDALIST trial).

Luspatercept has been recently approved for patients with very-low-risk, low-risk, or intermediate-risk MDSs with RS, who had an unsatisfactory response to or are ineligible for ESA therapy based on the MEDALIST trial, a double-blind, placebo-controlled, phase 3 trial [34]. Two hundred twenty-nine patients were randomized to either luspatercept or placebo. TI for 8 weeks or longer, following IWG 2006 criteria, was observed in 38% of the patients in the luspatercept group, as compared with 13% of those in the placebo group (*p* = 0.001). Higher erythroid responses were also observed (53% vs. 12%), with a median duration of RBC-TI of 30.6 weeks and without a significant increase in toxicity or increased risk of transformation to AML. Longer follow-up is needed to better evaluate the impact of luspatercept in the disease prognosis. The COMMANDS trial (ClinicalTrials.gov, number NCT03682536) is currently studying the efficacy of luspatercept in ESA naïve subjects who require red blood cell transfusion.

Recommendation: Luspatercept has been recently approved for patients with LR-MDS with ring sideroblasts, who had an unsatisfactory response to or are ineligible for ESA.

#### 2.2.2. Hypomethylating Agents

Hypomethylating (HMA) agents are among the standards to treat high-risk MDSs and are extensively used in low-risk diseases in the US. However, they have not been approved in Europe for the latter indication. Their efficacy in low and intermediate-risk patients has been indicated in several studies [36,37], particularly for those patients that have failed or are not eligible for ESA and lenalidomide. A recent meta-analysis, including 223 low-risk transfusion-dependent patients from 6 clinical trials treated with azacitidine (AZA), showed 38.9% achieved RBC-TI [38]. A study with decitabine (DEC) (one dose versus daily dosage for three days) showed an overall improvement rate of 23%, with over 50% achieving RBC-TI and platelet transfusion independence in both groups [39].

Furthermore, a randomized phase II trial comparing low dose AZA versus low dose DEC for lower-risk MDS in 113 patients showed an overall response rate of 62%, with 37% of patients achieving a CR [40]. Both AZA and DEC showed high response rates (49% vs. 79%, *p* = 0.03) and cytogenetic response rates (61% vs. 25% *p* = 0.02), as well as a high percentage of patients achieving transfusion independence (32% vs. 16%). Median OS was not reached at 20 months of follow-up, and toxicity was acceptable with no clinically significant grade 3 Es. All of these data support using azanucleosides in treating lower-risk MDS patients that are unresponsive to growth factors and/or lenalidomide. However, to date, no clinical trials have demonstrated an impact on OS in low-risk patients treated with HMA. More studies are needed, focus on OS as the primary endpoint in patients with low-risk MDS.

Recommendation: AZA could be considered in treating patients with LR-MDS without response or after failure to ESA and patients with the presence of 5q deletion not responding to lenalidomide. However, given the lack of evidence on the impact of this treatment on survival and quality of life in these low-risk patients, its use requires an individualized risk/benefit assessment [12].

#### 2.2.3. Allogenic Stem Cell Transplantation

Allo-SCT is not recommended as frontline therapy for lower-risk MDS independent of age. The exception may be a subset of young patients with hypoplastic MDS [15]. In a first approach to the question of whether the anticipated early mortality of allo-SCT can be overcome by its potential benefit on survival, it was established that for low and intermediate-1 IPSS groups, delayed transplantation maximized OS. This survival advantage was greater in patients under 40 years [41].

The benefit on survival of allo-SCT may be clearer in those patients of younger age where sequential therapies, such as ESA, lenalidomide and HMA have failed and in the absence of available clinical trials. In this sense, allo-SCT has shown to significantly increase OS of low-risk MDS patients after failure of HMA compared to no further treatment, conventional chemotherapy or study drugs [36].

Recommendation: We generally do not recommend allo-SCT in patients with LR-MDS at initial presentation. However, it should be considered individually in young patients refractory to other treatments. Due to the difficulty of identifying a donor, we recommend referring young patients to a stem cell transplantation consult as soon as possible with the intention of having a donor identified for the future [12].

### 2.3. Emerging Strategies for Management LR-MDS

#### 2.3.1. Roxadustat

Roxadustat is an oral hypoxia-inducible factor (HIF) prolyl hydroxylase inhibitor that enhances erythropoiesis through HIF-mediated transcription. It increases endogenous erythropoietin production, stimulates EPO receptor synthesis and modulates hepcidin to allow for better iron absorption and tissular management [42]. After its apparent efficacy in treating anemia in over 400 patients with chronic kidney disease, it is currently being studied in a phase III trial for anemia in low-risk MDS patients. Preliminary results, including 24 LR-MDS patients treated in the open-label dose-finding phase, reported a 54% erythroid response rate with 38% transfusion independence after 28 weeks of therapy.

#### 2.3.2. Imetelstat

Imetelstat is a telomerase inhibitor under investigation for LR-MDS patients with high transfusion burden and ESA failure or serum EPO > 500 U/L. The IMerge study is a phase II/III study with Imetelstat 7.5 mg/kg intravenously in 28 day cycles. The phase II portion of the trial included 38 non-del(5q) patients and showed 68% hematological improvement with transfusion independence rates of 45% and 26% at 8 and 24 weeks, respectively [43]. The phase III portion of the trial, placebo-controlled, is currently ongoing.

Recommendation: Currently, these treatments should be reserved for use within a clinical trial.

## 3. High-Risk MDS (HR-MDS)

**Figure 1:**
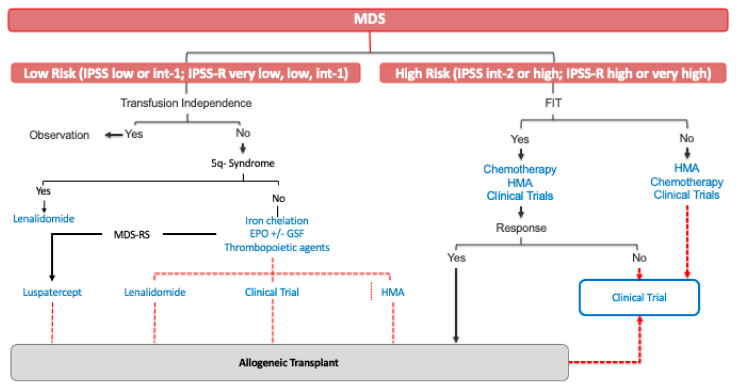
Current MDS treatment. MDS: myelodysplastic syndrome; int: intermediate; MDS-RS: a myelodysplastic syndrome with ring sideroblast; EPO: erythropoietin; HMA: hypomethylating agents.

Treatment of this subset of patients has substantially evolved during past two decades. Treatment of HR-MDS aims to change the natural history of the disease, prolonging OS and ultimately attempting to limit disease progression. Nowadays, the only curative treatment for HR-MDS is allogeneic transplantation. However, the mean age of presentation of MDS place most patients as not candidates for allogeneic transplantation (Figure 1).

### 3.1. Newly Diagnosed HR-MDS

#### 3.1.1. Hypomethylating Agents 

Azanucleotides play a crucial role in the frontline treatment of HR-MDS. AZA is, to date, the only agent approved in Europe based mainly on data from the CALGB 9221 and AZA-MDS001 clinical trials [44] (Table 2).

The CALGB study randomized 191 patients to receive AZA or BSC [45]. There were high rates of response in the AZA group (60% vs. 5%, *p* < 0.001), with a delayed median time to leukemic transformation or death (21 months vs. 13 months *p* = 0.007). The AZA-MDS001, a phase III clinical trial, randomized 175 patients to either AZA (75 mg/m^2^ per day for 7 days on 28 day cycles) or BSC (low-dose cytarabine or intensive chemotherapy) [46]. AZA showed prolonged median OS compared with conventional care regimens (24.5 months vs. 15.0 *p* = 0.0001). Of note, treatment with AZA also improved RBC transfusion dependency and rate of infections. The results from these studies have established treatment with AZA (75 mg m^2^ per day for 7 days on 28 day cycles) as the standard of care for HR-MDS patients. In a study conducted by the Groupe Francophone des Myelodysplasies (GFM), including 282 HR-MDS patients treated with AZA, adverse prognostic factors that independently predict shorter OS were poor-risk cytogenetics, performance status > 2, presence of circulating blasts, and red blood cell transfusion dependency [47]. Interestingly, prior treatment with cytarabine was also predictive of poorer OS.

Similar studies have been conducted to show the efficacy of DEC in these patients (Table 2). An initial trial randomized 170 patients to receive either DEC (135 mg/m^2^ per course repeated every 6 weeks) or BSC [48]. Overall, response rates were higher in the DEC group (17% vs. 0%, *p* = 0.001) with a median response duration of 10.3 months, which was associated with higher transfusion independent rates. Even if there was not a significant reduction in time to progression to AML, this study culminated with the approval of the drug in the US. It also has led to the conduction of new trials directed at establishing the optimal dosing regimens as well as confirming the efficacy and safety. Studies by the MDACC group and the ADOPT trial indicate an optimal dosage of 20 mg/m^2^ for 5 days every 28 days [49,50]. Of note, in the EU, DEC is not approved for patients with MDS, only in AML patients not eligible for standard induction chemotherapy.

ASTX727 is an oral agent that combines DEC with the cytidine deaminase inhibitor cedazuridine with a similar safety profile, dose-dependent demethylation and clinical activity when compared to parenteral DEC [51,52]. In view of these results, the FDA has approved ASTX727 to treat patients with high-risk MDS and CMML (IPSS intermediate-1 or higher).

HMA therapy should not be interrupted during the first 4–6 cycles in the absence of serious adverse events since premature interruption may lead to rapid loss of response.

Recommendation: AZA should be considered as the first-line treatment in not transplant-eligible HR-MDS. AZA is preferable to DEC as a hypomethylating agent in treating high-risk MDS since it has shown a substantial benefit in OS [12].

#### 3.1.2. AML-Like Chemotherapy

For many years, AML-like chemotherapy was the only therapeutic option for patients with HR-MDS. Intensive AML-like chemotherapy reported 50–60% CR rates. However, it also has a high incidence of early death (20–25%) with 20–25% refractory disease [57,58,59,60,61]. Long-term results leave little room for optimism with a very high risk of relapse (RR 70–80%), short duration of remission (median 8 months) and OS (median 12 months), and low proportion of patients surviving disease-free (10–20%).

OS with chemotherapy was significantly lower than that of a cohort of similar patients treated with DEC [62]. In addition, in a randomized trial, the median OS was 9 months lower than with AZA, although the differences were not significant due to the small number of patients who were candidates for intensive chemotherapy [46].

The most important prognostic factor of response to AML-like therapy is unfavorable karyotype and the presence of *TP53* mutations, with lower CR rates and short duration of response [63]. On the other hand, a recent retrospective study of 31 patients with MDS or CMML with *NPM1* mutation has observed better OS and EFS with AML-like chemotherapy and subsequent transplantation than with AZA (OS not reached versus 16 months, *p* = 0.047), suggesting a behavior most similar to AML in this patient subgroup [64].

Recommendation: The use of AML-like chemotherapy in high-risk MDS patients is in disuse. However, it should be considered in young patients, without comorbidities and with good prognosis cytogenetics (at least in those with NPM1 mutations).

#### 3.1.3. Allogenic Stem Cell Transplantation

Allo-SCT remains the only option for curation in MDS, and thus individual assessment of each patient’s eligibility for the procedure should be performed upon diagnosis. However, the proportion of patients that can benefit from this treatment is short, primarily because of age and comorbidities that go against performing a procedure with high associated mortality risk [15,41,65].

Allo-SCT can prolong disease-free survival in up to 30–50% of patients [66], but the applicability of allo-SCT remains restricted to a subset of younger patients with an optimal donor, where prolonged disease-free survival can overcome the toxicity of the treatment [15,67]. As cited above, two Markov decision models pointed towards improved survival with allo-SCT in HR-MDS patients [41,68]. Of note, several studies have reported poorer outcomes of allo-SCT in terms of OS on patients with high-risk genetic mutations, particularly in *RAS*, *TP53*, *RUNX1*, *ASXL1* and *JAK* [69,70].

The question rests upon the election of the most appropriate timing, the need and choice of the induction therapy and conditioning regimen. Early transplantation in high-risk eligible patients seems to be more beneficial [67,68]. Regarding whether or not allo-SCT should be preceded by a cytoreductive regimen (chemotherapy or HMA), many authors consider that when marrow blasts >10% at the time of transplantation, pre-transplant therapy is required because of the very high relapse risk post-transplant, but there are no prospective data on which is the best treatment to use as induction and HMA, or AML-like chemotherapy are used equally. Data comparing myeloablative regimens with reduced-intensity conditioning (RIC) therapy remains the subject of study. However, young fit patients appear to benefit more from myeloablative regimens, while in older patients, RIC appears to reduce therapy-related mortality, although assuming a higher risk of relapse [68,71].

Recommendation: All HR-MDS patients who are potential candidates for allo-SCT should be considered and referred to an allo-SCT consultation since starting treatment. In HR-MDS patients, allo-SCT should be performed as soon as an appropriate donor is located [12].

### 3.2. Future Therapies for HR-MDS: HMA-Based Combination Therapies 

#### 3.2.1. Azacitidine + Pevonedistat

Pevonedistat (TAK-924/MLN4924) is an inhibitor of the NEDD8-activating enzyme that has shown its efficacy as monotherapy in RR AML and also in combination therapy with AZA in this disease in phase 1b trials [72]. Based on this, a phase 2 trial of pevonedistat plus AZA versus AZA for higher-risk MDS/chronic myelomonocytic leukemia or low-blast AML was carried out and recently published [53]. Patients were randomized to either pevonedistat 20 mg/m^2^ (intravenous) on days 1/3/5, plus AZA 75 mg/m^2^ (intravenous or subcutaneous) on days 1–5/8–9, or AZA alone on the same schedule, in 28 day cycles (Table 2). Treatment was given until unacceptable toxicity, relapse, transformation to AML, progressive disease (PD), or the initiation of subsequent anti-cancer therapy or hematopoietic SCT. Efficacy favored pevonedistat plus AZA among all groups in terms of EFS (median 21.0 vs. 16.6 months; *p* = 0.076) compared with AZA alone. Results were particularly true for high-risk MDS, with a median OS of (23.9 vs. 19.1 months) and EFS (median 20.2 versus 14.8 months, *p* = 0.045). Patients with higher-risk MDS were more likely to achieve a response with the combination treatment compared AZA monotherapy (ORR 79.3% vs. 56.7%). CR rate was significantly higher for AZA with pevonedistat (51.7% vs. 26.7%), and what it is more important, duration of response was also impressively improved (median 34.6 versus 13.1 months). Lower transfusion rates were observed without higher rates of cytopenias. Median time to AML transformation was prolonged in high-risk MDS patients receiving the combination (10.6 vs. 7.88 months). Importantly, the efficacy of the combination was preserved in patients with high-risk cytogenetics and adverse risk mutations, including *TP53*. Overall, combining pevonedistat with AZA appears safe and effective in treating this subset of patients. A phase 3 trial is currently ongoing to back up these results (NCT03268954).

#### 3.2.2. Azacitidine + Magrolimab

CD47 is a “do not eat me signal” that allows for macrophage immune evasion by tumor cells of different cancers. Increased CD47 expression has been shown to predict a worse prognosis in AML patients [54]. Magrolimab is a first-in-class immune checkpoint inhibitor targeting CD47 that eliminates tumor cells through macrophage phagocytosis. It synergizes with azacitidine to induce remissions in AML xenograft models. Based on this, 5F9005 is a phase I study of the tolerability and efficacy of anti-CD47 antibody Magrolimab combined with AZA in MDS and AML patients (Table 2). Overall, response rate (ORR) of the combination was 91% (42% CR, achieving 56% at 6 months) [73]. The cytogenetic response was achieved in 35% of all responding patients with abnormal cytogenetics at baseline. The efficacy of the combination was also applied to TP53-mutant MDS (75% CR), although these patients constituted a small subpopulation of the study (4 MDS and 12 AML). In this sense, TP53 mutant VAF decreased significantly with treatment. Toxicity was acceptable, with no maximum tolerated dose reached and without clinically significant cytopenias, infections or immune-related Es. Of note, after the initial priming dose, there is a transient mild hemoglobin drop (mean 0.4 g/dL), with most patients presenting a significant hemoglobin improvement and decrease in transfusion dependency over time (RBC-TI of 58% in transfusion-dependent patients at baseline). Expansion cohorts are ongoing in MDS and AML (NCT03248479), with registrational studies in progress for MDS and planned for TP53-mutant AML.

#### 3.2.3. Azacitidine + Eprenetapopt

As with many other cancers, *TP53* mutations yield significantly worse prognoses and outcomes in MDS patients. Eprenetapopt (APR-246) is a methylated derivative of PRIMA-1, a drug that induces tumor cell apoptosis through the restoration of mutant p53 protein [74]. Preclinical studies have shown that eprenetapopt is efficient on its own, while it is also synergizing with AZA in TP53-mutated MDS and AML cell lines and in TP53-mutated cells from MDS and AML patients [75]. A phase Ib/II study to determine the safety and efficacy of eprenetapopt (APR-246) with AZA in patients with *TP53*-mutant MDS or AML with a low percentage of blasts has recently been published, with highly promising results [55] (Table 2). The ORR of the 51 patients enrolled (40 MDS) was 71%, with CR in 44%. In the MDS cohort, 73% responded, with 50% achieving CR and 58% obtaining a cytogenetic response. TP53 VAF was significantly reduced among responding patients, with 38% of patients achieving complete molecular remission defined as VAF under 5%. Responding patients achieved significantly prolonged OS (14.6 vs. 7.5 months, *p* = 0.0005). Toxicity was acceptable, with the most common grade 3 or more AEs being febrile neutropenia and cytopenias. The results of this study are consistent with the phase II study by de GFM evaluating the safety and efficacy of eprenetapopt in combination with AZA in untreated high or very high *TP53* mutated MDS and AML patients [76]. These studies have led to the ongoing pivotal phase III, multicenter, randomized study of eprenetapopt combined with AZA versus AZA alone in patients with *TP53*-mutant MDS (NCT03745716).

#### 3.2.4. Azacitidine + Venetoclax

BCL2 is an anti-apoptotic protein that is over-expressed in several tumors, including MDS. Venetoclax is a selective small-molecule BCL2 inhibitor that can induce apoptosis in tumor cells that are dependent on BCL2 for survival [56]. The association of Venetoclax and AZA has shown efficacy in frontline therapy of older patients with AML [77]. An ongoing phase 1b, open-label, multicenter study (NCT02966782), is evaluating the safety and efficacy of Venetoclax monotherapy compared to venetoclax plus azacitidine combination therapy with extremely promising results (Table 2). The 28 day cycles consisted of AZA 75 mg/m^2^ days 1 through seven, plus venetoclax 400 mg on days one through 14. Data from this study in terms of efficacy and patient-reported outcomes were presented at the ASH 2019 and 2020 meetings. There was an ORR of 77% in a cohort of previously untreated high-risk patients, with 37% of these achieving CR. In these patients achieving CR reported maintained improvement in dyspnea and fatigue and a moderate to large improvement in QoL. Survival estimates are 59.6% at 24 months. The most frequent adverse event was the presence of cytopenias. Overall, the combination treatment seems effective and safe, and thus there is an ongoing phase III trial to evaluate its role in newly diagnosed high-risk MDS patients (VERONA trial, NCT04401748).

Recommendation: Currently, these treatments should be reserved for use within a clinical trial

### 3.3. Hypomethylating Agent Failure in HR-MDS

Despite approximately 50% responses with HMA and improved overall survival, the response time with these therapies is often less than two years. Patients with relapsed or refractory higher-risk MDS have a median OS of fewer than six months, and there are no approved second-line treatments for this subset of difficult-to-treat patients [78,79]. The incorporation of molecular studies has been shown to improve prognostic stratification in these patients [4]. Allo-SCT needs to be considered in this scenario, but a retrospective study showed that HMA failure is associate with a higher risk of post allo-SCT relapse [67]. In this sense, some strategies, such as haploidentical NK-cell therapy, have demonstrated their role as a bridge to allo-SCT in patients with high-risk MDS and HMA failure [80]. However, in those patients, who are not candidates for allo-SCT, the only possibility available outside of a clinical trial is to change HMA, assuming a modest ORR of 19% [81]. New agents are being evaluated, with the most promising results shown by combination therapies based on HMA.

#### 3.3.1. Rigosertib

Rigosertib is a multikinase inhibitor that ultimately acts upon the de PI3K pathway, thus inducing inactivation of the different proteins it targets. Two hundred ninety-nine patients were enrolled at the ONTIME trial and randomized to rigosertib or best supportive care. The trial failed to show an effect on OS that favored rigosertib (8.2 months vs. 5.9, *p* = 0.33), with cytopenias being the most frequent therapy-related AE [82]. However, these results have led to a subsequent study of rigosertib’s role on patients primarily resistant to HMA and very high-risk patients (NCT02562443, INSPIRE trial).

#### 3.3.2. Immune Checkpoint Inhibitors

Immune checkpoint inhibitors targeting programmed death1 (PD1) and its ligand (PDL1) or cytotoxic T lymphocyte-associated protein 4 (CTLA-4) have been explored in the context of HMA failure high-risk MDS. A phase 2 clinical trial has tested nivolumab and ipilimumab in MDS patients after HMA failure showing some responses [83]. 2 out of 15 (13%) patients with HMA failure treated with nivolumab had response buy none of them achieved CR, while among 20 patients treated with ipilimumab, 7 (35%) had a response with 3 patients achieving CR (15%). Given their low single-agent activity, combination regimens could be more successful.

#### 3.3.3. Venetoclax

A phase I trial of venetoclax and azacitidine in higher-risk HMA failure MDS was presented at the 2019 ASH Annual Meeting [84]. Forty-six patients were enrolled in the trial, 22 in the venetoclax monotherapy arm and 24 patients in the venetoclax azacitidine combination arm. 80% of the patients have received one prior therapy before enrollment in the study. Among 16 patients evaluable for response in the monotherapy arm, 1 responded (7%), and stable disease was observed in 75% (12/16) patients. Median progression-free survival was 3.4 months with an estimated 6 month OS of 57%. The combination cohort showed encouraging results, with an ORR of 50% (12/24 patients), including 13% (3/24) CR and 38% (9/24) mCR. Stable disease was observed in 31% (10/24) patients. Median progression-free survival and OS were not reached with a 9 month estimate for OS of 83% (95% CI: 55%, 95%).

Recommendation: Since there is no approved treatment for hypomethylating-refractory patients, all HMA failure patients should be included in a clinical trial.

## 4. Conclusions

The heterogeneity of MDS results in significant challenges in developing new therapies. However, the treatment landscape to treat MDS patients is changing. Luspatercept was the first agent approved for MDS since 2006, followed by a new oral HMA, ASTX727. Figure 1 shows the current approach for MDS treatment in 2021.

For the first time, we have several treatments with different mechanisms of action that are effective in patients with MDSs. In the low-risk scenario, roxadustat and imetelstat have the potential to improve anemia in patients that have failed to ESA. However, the real change is happening in the high-risk patient. On one hand, to date, we have at least four AZA-based combinations that in preliminary studies have demonstrated their efficacy with an acceptable safety profile. However, more studies are needed to understand which subgroups of patients benefit from some treatment combinations more than others. Furthermore, the approval of a new oral HMA opens the door to purely oral treatment of HR-MDS, both in monotherapy and in combination, with what this may mean in terms of quality of life for these patients.

Unfortunately, HMA failure remains an unmet clinical need, and more clinical studies are needed to deal with this challenging scenario.

## Figures and Tables

**Table 1:** Revised international prognostic scoring system for myelodysplastic syndromes (2).

	0	0.5	1	1.5	2	3	4
Karyotype *	Very good		Good		Intermediate	Poor	Very poor
BM blasts (%)	0–2		3–4.9		5–10	>10	
Hemoglobin (g/dL)	≥10		8–9.9	<8			
Platelets (×10^9^/L)	≥100	50–99	<50				
ANC (×10^9^/L)	≥0.8	<0.8					

* Karyotype: very good: -Y, del(11q); good: normal, del(5q), del(12p), del(20q), double, including del(5q); intermediate: del(7q), +8, +19, i(17q), any other single or double independent clones; Poor: −7, inv(3)/t(3q)/del(3q), double, including −7/del(7q), complex: 3 abnormalities; very poor: complex: >3 abnormalities.

**Table 2:** Results from selected trials with hypomethylating agents in newly diagnosed high-risk MDS. AZA: azacitidine; DEC: decitabine; BSC: best standard of care; CR: complete response; LFS: leukemia-free survival; EFS: event-free survival; DoR: duration of response; OS: overall survival; iv: intravenous; sc: subcutaneous.

	Autor (Reference)	Therapy	Phase	N	Outcomes
**Monotherapy**	Silverman et al., 2002 [45]	AZA vs. BSC	III	191	ORR: AZA 60% vs. BSC 5% (*p* > 0.001) CR: AZA 7% vs. BSC 0% (*p* = 0.01) LFS: AZA 21 months vs. BSC 13 months (*p* = 0.007)
	Fenaux et al., 2009 [46]	AZA vs. BSC	III	358	ORR: AZA 29% vs. BSC 12% (*p* = 0.0001) CR: AZA 17% vs. 8% (*p* = 0.015) OS: AZA 24.5 months vs. BSC 15 months (*p* = 0.0001)
	Kantarjian et al., 2006 [48]	DEC vs. BSC	III	170	ORR: DEC 17% vs. BSC 0% (*p* = 0.001) CR: DEC 9% vs. BSC 0% LFS: DEC 12.1 months vs. BSC 7.8 months (*p* = 0.16) OS: DEC 14.0 vs. BSC 14.9 (*p* = 0.636)
	Kantarjian et al., 2007 [49]	DEC (20 mg/m^2^ iv × 5 days vs. 20 mg/m^2^ sc × 5 days vs. 10 mg/m^2^ iv × 5 days)	II	95	ORR: 73% CR: 34% 18 month EFS: 51% 18 month OS: 56%
	Steensma et al., 2009 [50]	DEC	II	99	ORR: 32% CR: 17% OS: 19.4 months
**Combination therapies**	Sekeres et al., 2021 [53]	AZA +/− pevonedistat	II	120	ORR: AZA + pevonedistat 79.3% vs. AZA 56.7% CR: AZA + pevonedistat 51.7% vs. AZA 26.7% DoR: AZA + pevonedistat 34.6 months vs. AZA 13.1 months EFS: AZA + pevonedistat 20.2 months vs. AZA 14.8 months (*p* = 0.045) OS: AZA + pevonedistat 23.9 months vs. AZA 19.1 months (*p* = 0.240)
	Sallmann et al., 2020 [54]	AZA + magrolimab	I	33	ORR: 91% CR: 42% DoR: median not reached OS: median not reached
	Sallman et al., 2021 [55]	AZA + eprenetapopt	Ib/II	40 (*TP53* mut)	ORR: 73% CR: 50% DoR: 8.4 months OS: 10.4 months
	Garcia et al., 2020 [56]	AZA + venetoclax	Ib	78	ORR: 77% CR: 37% DoR: 12.9 moths OS: 27.5 months
